# Human Pathogens in Body and Head Lice

**DOI:** 10.3201/eid0812.020111

**Published:** 2002-12

**Authors:** Pierre-Edouard Fournier, Jean-Bosco Ndihokubwayo, Jo Guidran, Patrick J. Kelly, Didier Raoult

**Affiliations:** * Université des la Méditerranée, Marseille Cedex, France; † Centre Hospitalier Universitaire, Bujumbura, Burundi; ‡ Médecins sans frontière, Marseille, France; § Ross University, St. Kitts, West Indies

**Keywords:** body louse, Rickettsia prowazekii, Bartonella quintana, Borrelia recurrentis

## Abstract

Using polymerase chain reaction and sequencing, we investigated the prevalence of Rickettsia prowazekii, Bartonella quintana, and Borrelia recurrentis in 841 body lice collected from various countries. We detected R. prowazekii in body lice from Burundi in 1997 and in lice from Burundi and Rwanda in 2001; B. quintana infections of body lice were widespread. We did not detect B. recurrentis in any lice.

The body louse, Pediculus humanus corporis, is the vector of three human pathogens: Rickettsia prowazekii, the agent of epidemic typhus; Borrelia recurrentis, the agent of relapsing fever; and Bartonella quintana, the agent of trench fever, bacillary angiomatosis, endocarditis, chronic bacteremia, and chronic lymphadenopathy (1). Louse-borne diseases can be associated with high incidence of disease and death, especially epidemic typhus and relapsing fever, which can be fatal in up to 40% of patients (2). The diseases are mostly prevalent in people living in poverty and overcrowded conditions, for example, homeless people and those involved in war situations (2).

Epidemic typhus, trench fever, and relapsing fever have been the subject of many studies, most of which were conducted between World War I and the 1960s. However, medical interest in the diseases and lice waned for almost 30 years. Since 1995 louse-borne diseases have had a dramatic resurgence, and trench fever has been diagnosed in many countries including the USA (3), Peru (4), France (5), Russia (6), and Burundi (7). In 1997 the largest outbreak of epidemic typhus since World War II occurred in Burundi among refugees displaced by civil war (7). A small outbreak also occurred in Russia (8), and evidence of R. prowazekii infection in Algeria was provided (9).

At the Unité des Rickettsies, we developed a polymerase chain reaction (PCR) assay to survey for human pathogens transmitted by the parasites; the assay can detect as few as 1–20 copies of the DNA of R. prowazekii, B. quintana, and Borrelia recurrentis in body lice (10). In 1995, we found R. prowazekii–positive lice in inmates of a Burundi jail (11), which was the source of a major outbreak of epidemic typhus in the country in 1996 (12). In 1997, we investigated an outbreak of pediculosis in refugee camps in Burundi. We identified R. prowazekii and B. recurrentis in body lice and epidemic typhus and trench fever in refugees (7,10). From April 1997 to December 1998, after our reports, a new strategy was designed to control typhus and trench fever. Health workers treated any patient with fever >38.5°C with a single dose of doxycycline (200 mg), a drug highly effective in the treatment of typhus (7). The program proved extremely successful, and in a follow-up in 1998 (10) we did not detect R. prowazekii in body lice collected in refugee camps in the country (Table 1).

**Table 1:** Prevalences of infections in body lice collected in various areas of the world

				Detection^a^ of	
Country	Source, yr	Reference^b^	No.	*Rickettsia prowazekii* (no., %)	*Bartonella quintana* (no., %)
Body lice					
France	Homeless in Marseille, 1998–2001	PS^c^	324	0	32 (9.9%)
France	Homeless shelter in Marseille, 2000	(13)	161	0	42 (26.1%)
France	Isolated homeless in Marseille, 1998	(10)	75	0	3 (4.0%)
The Netherlands	Homeless in Utrecht, 2001	PS	25	0	9 (36.0%)
Russia	Homeless in Moscow, 1998	(10)	268	0	33 (12.3%)
Tunisia	Homeless in Sousse, 2000	PS	3	0	0
Algeria	Homeless in Batna, 2001	PS	33	0	0
Congo	Refugee camp, 1998	(10)	7	0	0
Burundi	During typhus outbreak				
	Jail, 1997	(10)	10	2 (20%)	0
	Refugee camp, 1997	(10)	63	22 (35%)	6 (9.5%)
	After typhus outbreak				
	Refugee camp, 1998	(10)	91	0	13 (14.3%)
	Refugee camp, 1998	PS	38	0	8 (21.0%)
	Refugee camp, 2000	PS	111	0	100 (90%)
	Refugee camp, 2001	PS	33	7 (21%)	31 (93.9%)
Rwanda	Jail, 2001	PS	262	19 (7%)	6 (2.3%)
Zimbabwe	Homeless in Harare, 1998	(10)	12	0	2 (16.7%)
Australia	Homeless in , 2001	PS	2	0	0
Peru	Andean rural population	(10)	73	0	1 (1.4%)
Peru	Andean rural population	PS	10	0	0
Head lice					
France	Schoolchildren	PS	20	0	0
Portugal	Schoolchildren	PS	20	0	0
Russia	Schoolchildren	PS	10	0	0
Algeria	Schoolchildren	PS	18	0	0
Burundi	Schoolchildren	PS	20	0	0
China	Schoolchildren	PS	23	0	0
Thailand	Schoolchildren	PS	29	0	0
Australia	Schoolchildren	PS	3	0	0

^a^*Borrelia recurrentis* could not be detected in any of the tested lice. ^b^Data previously reported in the indicated reference. ^c^PS, present study.

Since 1998, we have continued our efforts and have collected 841 body lice obtained by medical staff from our laboratory or local investigators in Burundi, Rwanda, France, Tunisia, Algeria, Russia, Peru, China, Thailand, Australia, Zimbabwe, and the Netherlands (Table 1). In Burundi, lice were collected during the outbreak of epidemic typhus and on three occasions (1998, 2000, and 2001) after the outbreak had been controlled. Lice found on any part of the body, except the head and pubis, were regarded as body lice. The lice were transported to France in sealed, preservative-free, plastic tubes at room temperature. Delays between collection and analysis ranged from 1 day to 6 months. As negative controls, we used specific pathogen-free laboratory-raised body lice (Pediculus humanus corporis strain Orlando). To prevent contamination problems, as positive controls we used DNA from R. rickettsii R (ATCC VR-891), Bartonella elizabethae F9251 (ATCC 49927), and Borrelia burgdorferi B31 (ATCC 35210), which would react with the primer pairs we used in our PCRs but give sequences distinct from the organisms under investigation. To prevent false-positive reactions from surface contaminants, each louse was immersed for 5 min in a solution of 70% ethanol–0.2% iodine before DNA extraction and then washed for 5 min in sterile distilled water. After each louse was crushed individually in a sterile Eppendorf tube with the tip of a sterile pipette, DNA was extracted by using the QIAamp Tissue Kit (Qiagen, Hilden, Germany), according to the manufacturer’s instructions. This kit was also used to extract DNA from the organisms cultivated in our laboratory under standard conditions to be used as positive controls. The effectiveness of the DNA extraction procedure and the absence of PCR inhibitors were determined by PCR with broad-range 18S rDNA-derived primers (10).

To detect louse-transmitted pathogens, we used each of the genus-specific primer pairs described in Table 2 in a separate assay. A total of 2.5 μL of the extracted DNA was used for DNA amplification as previously described (10). PCRs were carried out in a Peltier Thermal Cycler PTC-200 (MJ Research, Inc., Watertown, MA). PCR products were resolved by electrophoresis in 1% agarose gels. All lice yielded positive PCR products when amplified with the 18S rRNA-derived primers, demonstrating the absence of PCR inhibitors. Negative controls always failed to yield detectable PCR products, whereas positive controls always gave expected PCR products. PCR amplicons were purified by using the QIAquick Spin PCR purification kit (Qiagen) and sequenced using the dRhodamine Terminator cycle-sequencing ready reaction kit (PE Applied Biosystems, Les Ulis, France), according to the manufacturer’s recommendations. Sequences obtained were compared with those in the GenBank DNA database by using the program BLAST (14).

**Table 2:** Oligonucleotide primers used for PCR amplification and sequencing^a^

Primer (reference)	Nucleotide sequence	Organism or sequence used	Size of expected PCR product (bp)
CS-877 (10)	GGG GGC CTG CTC ACG GCG G	*Rickettsia* species	396
CS-1273 (10)	ATT GCA AAA AGT ACA GTG AAC A	*Rickettsia* species	
QHVE1 (10)	TTC AGA TGA TGA TCC CAA GC	*Bartonella* species	608
QHVE3 (10)	AAC ATG TCT GAA TAT ATC TTC	*Bartonella* species	
Bf1 (10)	GCT GGC AGT GCG TCT TAA GC	*Borrelia* species	1,356
Br1 (10)	GCT TCG GGT ATC CTC AAC TC	*Borrelia* species	
18saidg (10)	TCT GGT TGA TCC TGC CAG TA	Arthropods	1,526
18sbi (10)	GAG TCT CGT TCG TTA TCG GA	Arthropods	

^a^PCR, polymerase chain reaction.

The sequences of the DNA amplicons we obtained were identical to those of R. prowazekii and B. quintana in GenBank. We detected R. prowazekii in body lice collected in Burundi in 2001 but not in those collected in 1998 and 2000, although they were positive for B. quintana. R. prowazekii was also detected in 7% of lice collected in Rwanda. We found B. quintana in body lice collected in France, the Netherlands, Russia, Burundi, Rwanda, Zimbabwe, and Peru. No PCR products were obtained for any of the lice when primer pair Bf1-Br1 was used, indicating lack of infections with Borrelia recurrentis.

Our PCR may greatly facilitate the study of lice and louse-borne diseases as it can be used to survey lice for these organisms, detect infected patients, estimate the risk for outbreaks, follow the progress of epidemics, and justify the implementation of controls to prevent the spread of infections. We have successfully applied the PCR assay to lice from homeless and economically deprived persons in inner cities of developed countries and found high prevalences of Bartonella quintana infections (3,5,6). Furthermore, we have emphasized the risk of R. prowazekii outbreaks in Europe, based on our findings of an outbreak of epidemic typhus in Russia, a case of Brill-Zinsser disease in France (15), and a case of epidemic typhus imported from Algeria (9).

The PCR assay on lice may help detect outbreaks. In recent epidemics of louse-borne infections, the prevalence of body louse infestations in persons has reached 90% to 100% before clinical signs of louse-borne disease were noted in the population (16). Experience has shown that the emergence and dissemination of body lice can be very rapid when conditions are favorable (17). In Central Africa, large outbreaks of lice infections occurred during civil wars in Burundi, Rwanda, and Zaire (16) and preceded the outbreak of epidemic typhus by 2 years (7). We clearly demonstrate the potential for further outbreaks of louse-borne diseases in Africa. Although lice from Burundi were negative for R. prowazekii in 1998 and 2000 as a result of the administration of doxycycline to patients, the persistence of the vector enabled the spread of R. prowazekii from human carriers back into the louse population. In 2001, we found that 21% of lice from refugee camps in the same areas of Burundi as sampled earlier were positive by PCR for R. prowazekii. Further samples submitted to our laboratory indicate a typhus outbreak is currently developing in refugee camps in Burundi (unpub. data). We also found R. prowazekii in 7% of body lice collected in 2001 from a jail in Rwanda. That the country is now host to 300,000 refugees from the January 2002 eruption of the Nyiragongo volcano is thus a concern.

Although lice from the other areas studied were free from typhus, we found B. quintana to be widely distributed; it was detectable in lice from France, the Netherlands, Burundi, Zimbabwe, and Rwanda. We could not find the organism in lice from Australia, Tunisia, and Algeria, but only small numbers of lice from these areas were studied. As with R. prowazekii, chronic bacteremia occurs with B. quintana infection in humans; the only way to eradicate the organism is to eliminate body lice. We were not able to detect Borrelia recurrentis in any of the lice, which indicates that infection rates with this organism are very low or the agent is restricted to specific geographic zones.

Our study has demonstrated the usefulness of PCR of body lice in ongoing surveillance of louse-associated infections. When faced with outbreaks of body lice or to follow-up outbreaks of louse-borne infections, investigators should consider using PCR for R. prowazekii, Bartonella quintana, and Borrelia recurrentis in body lice collected from the study area and shipped to their laboratories. Our results from Burundi highlight the necessity for using combinations of methods to control body lice and hence R. prowazekii infections.
